# Supplementary material for: Coordinated Emergence of Hippocampal Replay and Theta Sequences during Post-natal Development
Source: Curr Biol. 2019 Mar 4;29(5):834–840.e4. doi: 10.1016/j.cub.2019.01.005 (PMC6408330; doi:10.1016/j.cub.2019.01.005)
Supplement: Document S1. Figures S1–S4 [file mmc1.pdf]

Current Biology, Volume *29*

Supplemental Information

Coordinated Emergence of Hippocampal Replay and Theta Sequences during Post-natal Development

Laurenz Muessig, Michal Lasek, Isabella Varsavsky, Francesca Cacucci, and Thomas Joseph Wills

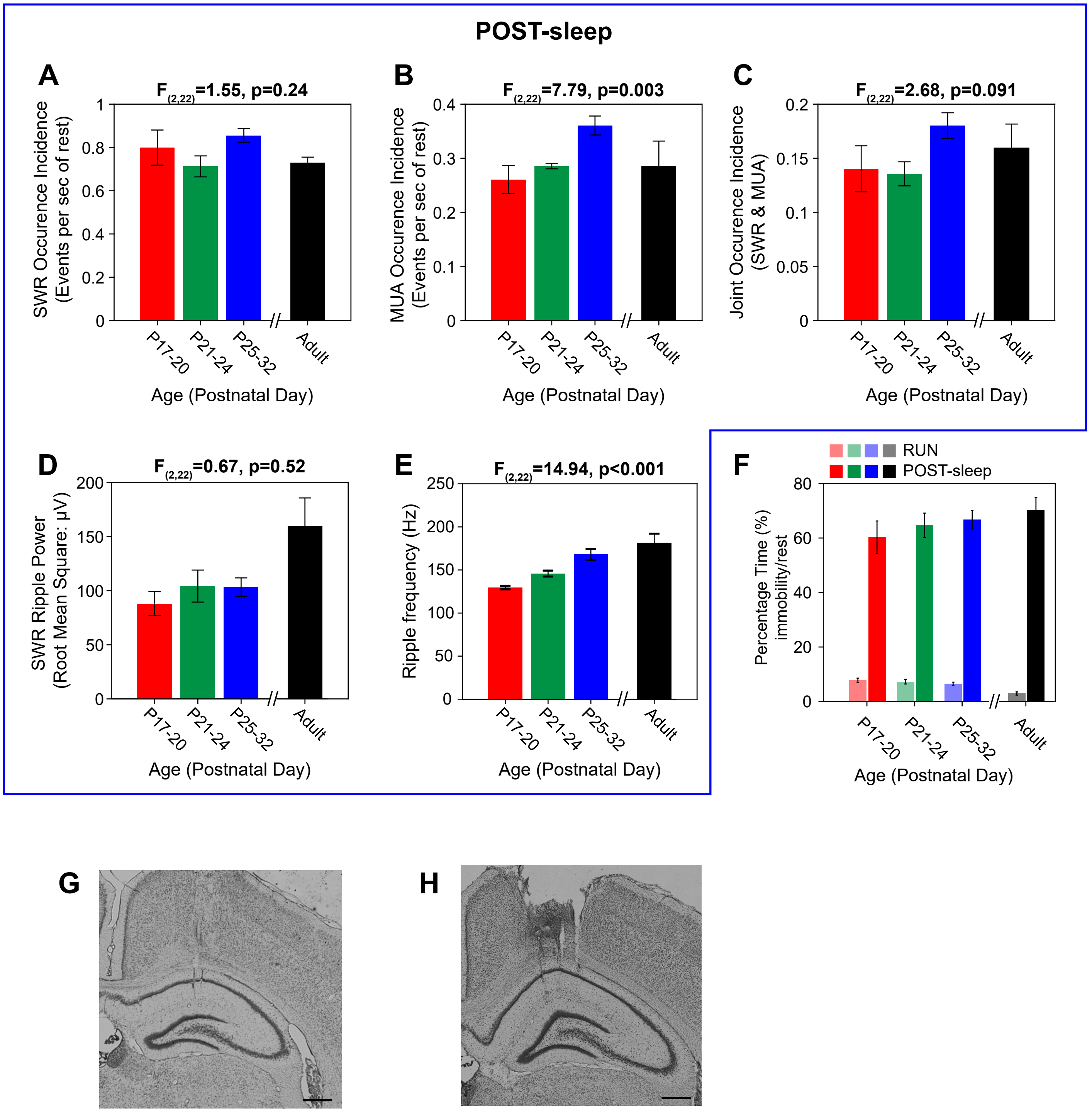

Figure S1. Characterisation of SWRs and MUA bursts during development. Related to figure 3. **(A-C)** Bar charts showing the mean incidence (±SEM) of the occurrence of SWRs (A), MUAs (B) and overlapping SWR/MUA events (C), in each experimental session. All y-axes show events per second of rest. 1-way ANOVA statistics comparing developing rat groups shown on top of graph. There is a significant increase in the occurrence of MUAs during development, but no significant change in the occurrence of SWRs, or overlapping SWR/MUA events. **(D)** Bar chart showing the average root-mean-square power (±SEM) of SWRs in each experimental session. There is no significant change in SWR power during development (1-way ANOVA statistics comparing developing rat groups shown on top of graph), though the power of adult SWRs is greater than that of those in developing rats. **(E)** Bar chart showing the mean ripple frequency (±SEM) of SWRs in each experimental session. There is a significant increase in ripple frequency during development: 1-way ANOVA statistics comparing developing rat groups shown on top of graph. **(F)** Bar chart showing the percentage trial time (±SEM) spent in non-locomotory bouts during RUN and in rest during POST-sleep. **(G-H)** Histological sections from two rats. Electrodes were implanted at P15 (G) and at P22 (H). Several microelectrode penetration tracks are visible, all coursing through the hippocampal CA1 pyramidal layer (scale bar 500 um).

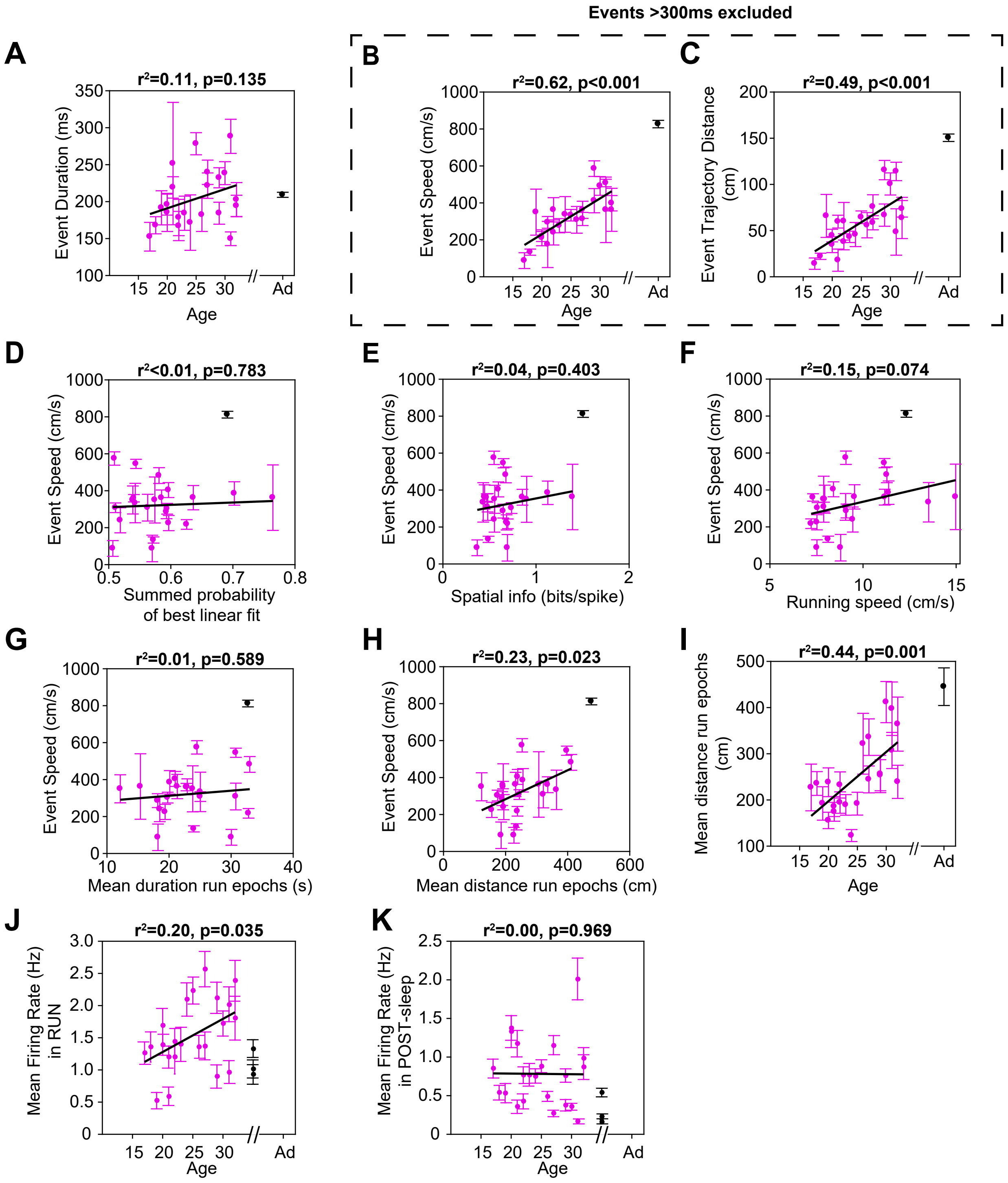

Figure S2. Developmental increases in linear trajectory speed of significant events are not due to changes in event duration, changes in place cell tuning, running speed, linear fitting or mean firing rate. Related to figure 3. In all panels each pink data point indicates the mean of a characteristic of significant linear events of one developing rat at one age point (±SEM). Adult event mean is shown in black, and is excluded from correlations. Correlation statistics are shown above plots. **(A)** No significant correlation between average MUA event duration and age of animals. **(B-C)** Furthermore, increases in mean trajectory speed (B) and distance (C) within each rat remain significantly correlated with age, even after events of >300ms are removed. **(D)** No significant correlation between the mean goodness of the linear fit (defined as the summed probability within the linear fit band) and the mean speed of linear trajectory events. **(E)** No significant correlation between mean linear trajectory event speed and the spatial tuning of complex spike cells (defined as the mean spatial information of the rate maps of the recorded ensemble, during exploration). **(F)** Trend for a significant correlation between mean linear trajectory event speed and the running speed of the rat (defined as the median running speed for the session, after exclusion of periods of immobility), as both these increase with age. The partial correlations of age versus event trajectory distance and speed, after controlling for running speed, remain significant (Distance r^2=0.49, p<0.001, speed r^2=0.52, p<0.001). **(G)** No significant correlation between the mean temporal duration of epochs of constant running direction in RUN sessions, and the mean speed of significant linear trajectories. **(H-I)** Significant correlation between mean distance traversed during epochs of constant running direction and the mean speed of significant linear trajectory events (H) as both scores increase with age (see (I) for scatterplot of mean distance traversed during epochs of constant running and age). However, the partial correlation for age versus distance and speed, after controlling for epoch distance, remain significant (Distance r^2=0.22, p=0.033, speed r^2=0.42, p=0.001). **(J-K)** Mean firing rate during RUN increases significantly with age, whilst mean firing rate during rest (POST-Sleep) remains unchanged across age. The increase in mean firing rate in RUN does not explain the increase in replay speed with age: the partial correlation between replay speed and age, controlling for mean rate in RUN, is significant (r^2=0.48, p<0.001). Finally, the partial correlation between replay speed and age remains significant when all of those covariates significantly changing with age (distance run in constant direction epochs, online decoding error [see S3], mean rate during RUN) are included as controlling variables (r^2=0.31, p=0.013).

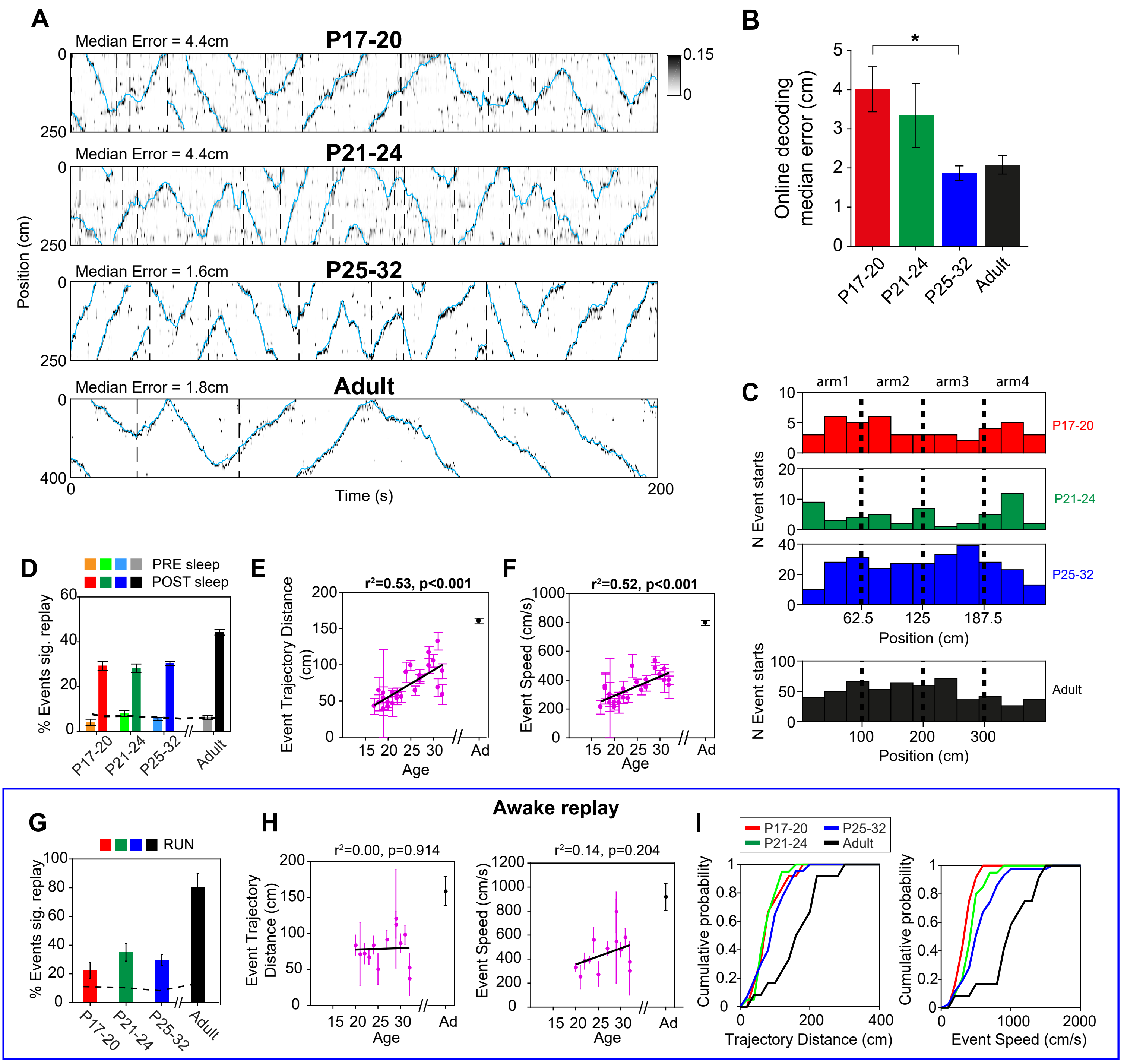

Figure S3. Developmental increases in linear trajectory speed are not due to general decoding error or shuffling method (A-F). Characterisation of awake replay development (G-I). Related to figure 3. (A) Bayesian decoding of position on the basis of CS cells spatially tuned firing in developing rats (decoding the position of the rat during RUN using RUN spiking). Posterior probabilities of position given spiking are shown in greyscale (black shows high probability). The x-axis shows time (total 200 secs), the y-axis shows position on the track (in cm). Pale blue line shows rat's camera recorded position. Immobility periods (defined as running speed <2.5cm/sec) were removed from analysis (indicated by vertical dashed lines). **(B)** Overall mean (±SEM) median decoding errors for each age bin. There is a significant decrease in online decoding error with age (ANOVA, $F_{2,19}$ = 4.61, p=0.023), but the decrease in decoding error does not explain the increase in replay speed with age (partial correlation of replay speed with age controlling for decoding error: r^2 = 0.41, p=0.002). Median decoding error for the whole trial is shown above the plot. **(C)** Significant linear trajectory events start at positions equally distributed throughout the square track. Histograms show counts of significant events with trajectories starting at corresponding points on the square track. At all ages, trajectories show no apparent bias to start at particular positions on the track. **(D-F)** Gradual emergence of rest replay between P17 and P32: results are unchanged when significance of event linear fit is determined by comparison to a population of shuffled events generated by randomly shifting spatial firing rate maps before decoding ('map shuffle'). In all of (D-F) each pink data point indicates the mean of a characteristic of significant replay events of one developing rat at one age point (±SEM). Adult event mean is shown in black, and is excluded from correlations. Correlation statistics are shown above the plots. **(D)** Bar charts showing the percentage of spiking events determined to contain significant linear trajectories following the map shuffle, in both PRE- and POST-sleep, at all ages. Bars show the 95% confidence intervals of the percentages, the dashed line indicates the 95% confidence level. Note that more events in developing rats are classified as containing significant linear trajectories, compared to the cell identity shuffle. **(E-F)** Mean distance covered (E) and mean speed (F) of significant linear trajectory events when using the map shuffle to determine event significance. Bars show ±SEM of scores within rat. Both scores show significant correlations with age. **(G-I)** Emergence of awake replay between P17 and P32. **(G)** Percentages (±95 confidence interval) of events with a significant linear trajectory during immobility periods in RUN across development. Dotted line represents 95% confidence threshold. At all ages, more events than expected by chance showed a significant linear trajectory. Distance covered (left) and speed (right) of decoded trajectories. For both plots, each data point represents mean (±SEM) of all significant linear events in one experimental session (one rat/day). Adult data (in black) represent overall mean across all sessions. For each measure r^2 and p-values of linear regression over age is indicated above plots (adult data are excluded from regression analyses). **(I)** Cumulative distributions of the distance covered (left) and the speed (right) of all significant linear trajectory events, in the age groups P17-20, P21-24, P25-32 and in adult animals.

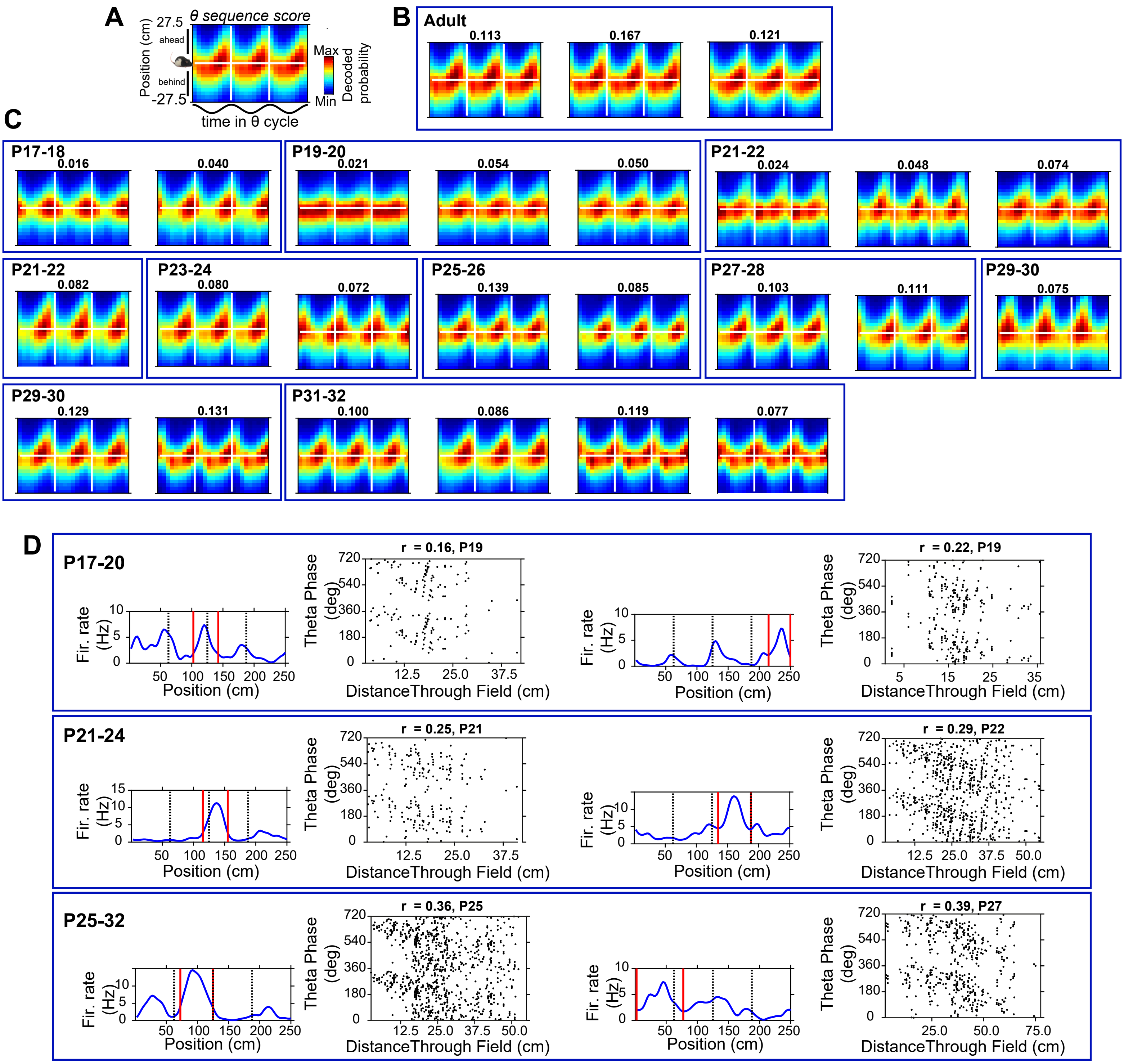

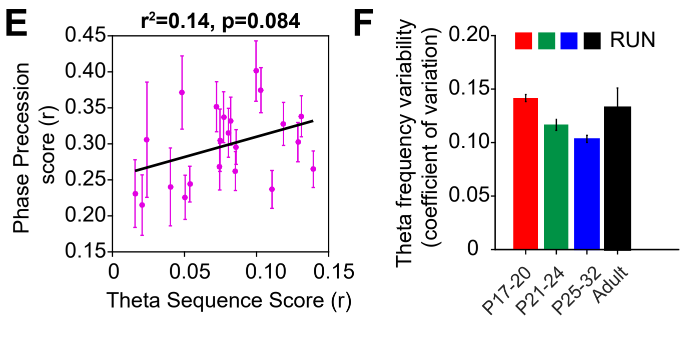

Figure S4. Development of theta sequences and phase precession. Related to figure 4. **(A)** Schematic example exemplifying format of posteriors displayed in (B, C). The probability posterior is derived from a single RUN session, where the x-axis shows the proportion of time elapsed during the theta cycle and the y-axis shows position on the track relative to the current location of the rat. The horizontal white line shows current rat location, the vertical white lines demarcate one theta cycle. Hot colours show high decode probabilities. Numbers above the plots show theta sequence score, defined as the circular-linear weighted correlation of the probability posterior. Theta sequences are indicated by a shift in the decoded position from behind to ahead of the rat within the theta cycle: this emerges gradually between P17 and P32. **(B)** Probability posteriors of all adult datasets. **(C)** Theta sequence emergence across development. Probability posteriors of all datasets are shown, ordered by age. **(D)** Examples of phase precession in six complex spikes cells recorded at different ages. Plots on left represent firing rate plotted against position on the track. Red lines indicate the main place field, in which phase precession was analysed. Plots on right show theta phase as a function of position within the main field. Numbers on top of plots are theta phase precession score (r - cicular linear correlation between theta phase and position on track) and age. Note the gradual advance of firing phase as the rat passes through each field, at all ages. Phase precession strength increases during development (linear regression of phase precession score over age in young rats; $r^2=0.31$, $p=0.007$). **(E)** Phase precession score is not significantly correlated with Theta sequence score. Each data point represents mean (±SEM) phase precession scores in one experimental session. **(F)** Significant decrease in Theta cycle length variability across development (one-way ANOVA of theta frequency coefficient of variation [standard deviation/mean] over age: $F(2,19)=20.7$, $P<0.001$; adult data are excluded from analysIs).
